# Do a pedagogical agent’s clothing and an animated video’s setting affect learning?

**DOI:** 10.3389/fpsyg.2023.1205338

**Published:** 2023-09-26

**Authors:** Daniela Decker, Martin Merkt

**Affiliations:** German Institute for Adult Education – Leibniz Centre for Lifelong Learning, Bonn, Germany

**Keywords:** pedagogical agent, adult learning, distance education, online learning, clothing, setting

## Abstract

Pedagogical agents are often used to enhance social cues in learning materials. The inclusion of pedagogical agents raises several design questions, for example on what kind of clothing the agent should wear. Further, it is not yet clear how the setting of an animated learning video (i.e., the digitally created background) affects learning. In an online experiment (*N* = 200), we investigated whether creating thematically appropriate clothing and setting has some added value in that it improves learning outcomes in comparison to more neutral assets. Whereas all participants acquired knowledge from the animated video, there were no main effects of clothing and setting for any of the dependent variables, but an interaction for learning outcomes ($\eta_{p}^{2}=0.02$), indicating that the appropriately dressed agent worked better combined with the inappropriate setting than with the appropriate setting. Overall, given those non-significant main effects and the small effect size of the interaction, there seem to be some degrees of freedom for designers of pedagogical agents in animated learning videos. However, these degrees of freedom may be limited to at least moderate (i.e., neutral) levels of appropriateness.

## 1. Introduction

Learning with (animated) online videos can take place in a variety of contexts, for example as additional learning material in formal education or in informal learning contexts, where learners choose learning content self-directed and decide for themselves how they want to learn with these materials (Lange, 2018). Given this increasing popularity among learners and the growing opportunities for educators to create these videos, it is important to understand how the decisions to design these materials affect learning. Regarding the increasing possibilities to include humanlike virtual characters into animated learning materials (i.e., pedagogical agents; Craig et al., 2002; Apoki et al., 2022), the design of such agents becomes another factor to consider in terms of its impact on learning. Whereas it is evident that the inclusion of a virtual pedagogical agent may serve as a social cue (Lester et al., 1997; Li et al., 2022), there are still some open questions regarding the specific design of the pedagogical agent as well as the content-appropriateness of the setting (i.e., the digitally created background) in that the pedagogical agent is integrated. In this manuscript, we will address whether designing thematically appropriate clothing and an appropriate setting for a pedagogical agent in an animated learning video benefits learners’ evaluations of the learning materials and learning outcomes. Before we go into detail about the empirical background regarding the pedagogical agent’s clothing and the setting of the learning materials, we will give a brief overview of potential effects of pedagogical agents in general.

### 1.1. The importance of social cues for learning

Influential theories such as the cognitive load theory (CLT) (Sweller et al., 1998; Sweller, 2011) and the cognitive theory of multimedia learning (CTML) (Mayer, 2014) still have a strong focus on the cognitive processes that underly successful learning. Most importantly, both theories share the assumption that learners’ cognitive resources are limited, and learning materials should be designed so that they do not result in cognitive overload. The CTML further states that learners must actively select, organize, and integrate incoming information to develop comprehensive mental representations in long-term memory. In such a purely cognitive approach, a pedagogical agent may be considered an interesting, but irrelevant addition to the learning materials, thus constituting a seductive detail that may increase extraneous cognitive load and thus hinder learning (for an overview, see Rey, 2012). However, extending these primarily cognitive theories, theories such as the social agency theory (Mayer et al., 2003; Mayer, 2014) emerged that emphasize the role of social cues in the learning process (also see social learning theory; Bandura, 1977). Further, the cognitive-affective-social theory of learning in digital environments (CASTLE; Schneider et al., 2022), which integrates a variety of theories on social cues, distinguishes between different types of social cues which may activate social schemas in learners resulting in increased (para-)social and metacognitive processes. These processes should subsequently affect the information selection, as well as the further processing in the working memory, which might also influence the building of mental models in long-term memory. In this regard, CASTLE (Schneider et al., 2022) emphasizes the use pedagogical agents as a means to provide both verbal and visual social cues. Therefore, including a pedagogical agent to provide social cues should have beneficial effects on learning due to an increased social response (Lester et al., 1997), which is reflected in increased brain activity during learning in areas of the brain that are usually associated with social processes (Li et al., 2022). Moreover, social cues provided by pedagogical agents might influence learning indirectly through factors such as motivation (Heidig and Clarebout, 2011; Bian, 2022; Sikström et al., 2022; Wang et al., 2023), perceived learning experience (Grivokostopoulou et al., 2020), and the perceived expertise of a pedagogical agent (Nguyen, 2023).

### 1.2. Use of pedagogical agents

Regarding the influence of pedagogical agents on recall, transfer, and motivation, Heidig and Clarebout (2011) suggested the pedagogical agents conditions of use (PACU) framework, due to the rather heterogenous findings in an analysis of 26 articles. The PACU framework includes, among others, considerations about agent design which distinguishes between the global design level (e.g., human vs. non-human, and static vs. animated), the medium design level (e.g., partial vs. full display, audio output, and language style), and the detail design level (e.g., age, gender, and clothing of the pedagogical agent).

Further reviews (Martha and Santoso, 2019; Sikström et al., 2022) and meta-analyses (Castro-Alonso et al., 2021) concluded that the inclusion of pedagogical agents facilitates learning with multimedia applications, even though the overall effects were rather small. However, in the studies analyzed by Castro-Alonso et al. (2021), there was significant heterogeneity regarding the observed effect sizes and moderation analyses revealed that effects of pedagogical agents were mostly moderated by characteristics of the studies (e.g., the design of the control group without the agent and type of randomization), but hardly by the agents’ design characteristics such as appearance (2D or 3D), non-verbal communication (gestures, gaze, and facial expressions), motion (static or animated), or voice (human or synthesized). On the one hand, this could be attributed to power problems because there were only few studies in some of the relevant categories for the moderation analyses, but it could on the other hand also hint toward the need to investigate other agent characteristics as potential moderators. In contrast to Castro-Alonso et al. (2021) and Dai et al. (2022) conclude that the success of pedagogical agents highly depends on the agents’ design, especially regarding the level of embodiment by means of gestures and facial expressions. However, the agent’s clothing was not included in the analysis. Further, Tao et al. (2022) focused on studies which covered different variations of agent design such as human likeness (from cartoon-like to photo-realistic) or gesturing. They concluded that an agent’s degree of human likeness and attractiveness could influence learning, but the results were mixed considering the question which agents are preferable. Again, the effects of the agents’ clothing were not considered. Finally, Liew and Tan (2021) focused their systematic review on the agent’s design, especially concerning cues for an agent’s expertise and emphasized the agent’s appearance, including clothing as one possible cue for expertise. With regard to clothing, they particularly emphasize that expertise cues could be conveyed if the pedagogical agent’s clothing reflects the social expectation of the clothing of an expert in their domain.

Since the appearance of a pedagogical agent may be linked to its perceived expertise, it might play an important role how learners typically imagine the appearance of a particular occupational group (Küster et al., 2019; Liew and Tan, 2021). In this regard, Veletsianos (2010) observed that the matching of an agent’s visual appearance to the teaching context affects learning outcomes. In a 2 × 2 design, they varied whether the agent was portrayed as a scientist or an artist by varying the characteristics of the face (neat short hairstyle without beard vs. mohawk with chin beard) and whether the agent talked about punk rock or nanotechnology. They found that the participants rated the artist agent as more knowledgeable than the scientist agent in the punk rock condition. However, both agents were rated as almost equally knowledgeable in the scientific nanotechnology domain. This finding may be due to stronger stereotypes regarding the facial appearance of a punk rocker compared to the facial appearance of a scientist, thus facilitating the visual manipulation in the punk rock domain. Further, Liew et al. (2013) also emphasized that for designing an expert-like agent, it is crucial how learners imagine experts in a field. Their manipulation of an expert-like agent vs. a peer-like agent was established by age and voice (calm vs. authoritarian). Although no significant effects of the agents’ expertise on learning outcomes were observed, female learners rated the expert-like agent as more trustworthy (Liew et al., 2013). Moreover, matching the appearance of a pedagogical agent with the associations triggered by the learning material, resulted in superior learning in an experiment (2 × 2) in that the age of the agent (young vs. old) was crossed with content relating to these age categories (relating to young vs. old age) (Beege et al., 2017).

In the current study, we investigated the effects of clothing as an attribute of pedagogical agents (detail design level, see Heidig and Clarebout, 2011). In particular, clothing which is typical for an expert in a field could influence the perceived expertise of a pedagogical agent (Baylor, 2011; Küster et al., 2019). Therefore, in the following section, we summarize research that focuses on the effects of clothing of both humans (e.g. Johnson et al., 2014; Beege et al., 2019) and pedagogical agents (Schmidt et al., 2019; Petersen et al., 2021).

### 1.3. Effects of clothing

In general, clothing is considered to be a form of communication that reveals information about the wearer (Damhorst, 1990). Following symbolic interactionism (Blumer, 1969), our behavior toward a person is influenced by their clothing and the meaning we attach to it.

In particular, clothing that is typically worn by members of a certain professional group (e.g., soldiers, policemen, doctors, postmen, judges, or priests) is often accompanied by an attribution of personal expertise (Kodžoman, 2019). Therefore, not only the perception of the competence often varies with a persons’ clothing (Gurney et al., 2017), but also the trust invested in them. For instance, Hartmans (2013) investigated the influence of a doctor’s clothing on patients’ trust and their perception of medical expertise. After viewing images of six models (three male and three female) in three age groups (25–35 vs. 35–50 vs. >50), each wearing five different outfits (leisure clothing vs. casual vs. semiformal vs. formal vs. professional), participants completed a questionnaire which revealed that patients trusted most in doctors wearing a white coat. Thus, the study shows that patients prefer doctors dressed in professional clothing.

In an educational context, the quality of a presentation given by formally dressed students was rated higher than that of casually dressed students (Gurung et al., 2014). Comparably, formally dressed teachers were perceived as knowledgeable, well prepared, and organized (Lukavsky et al., 1995), but less approachable than informally dressed teachers (Butler and Roesel, 1989). Further, Beege et al. (2019) investigated whether the clothing style (professional vs. non-professional) and the addressing style (frontal vs. lateral) of an instructor affect learning. In two experiments, the authors observed that professional clothing improved retention if the instructor in the video directly looked at the camera (frontal addressing style) (Beege et al., 2019). A more recent study by Beege et al. (2022) moreover emphasizes that the effects of the instructors’ professional appearance on learning and para-social interactions are enhanced when the instructor also communicates professionally.

Only few studies investigated the effect of pedagogical agents’ clothing. For example, Küster et al. (2019) observed that both a nurse outfit and a military outfit were associated with more perceived competence of an agent compared to a casual outfit. However, they used static pictures of the agents and did not include any measures of learning outcomes. In contrast, in a museum context, Schmidt et al. (2019) found positive effects on learning and presence, if an agent was represented as a museum guide rather than an astronaut. However, Petersen et al. (2021) observed heterogeneous results regarding the effects of the thematically appropriate agent design on learning in a virtual museum. Comparing an agent dressed as museum guide with the same agent displaying only black mesh and a control group without an agent, they found that all the investigated pedagogical agents seemed to hinder the acquisition of factual knowledge. In contrast, regarding conceptual knowledge, thematically appropriate agents had a positive effect on learning, but only if the agent did not show realistic features (i.e., no gestures, very limited idle animations, and no lip-syncing), and was presented in a rather static way.

In summary, research on the effects of clothing on learning outcomes is not yet conclusive and it is yet an open question how a pedagogical agent’s clothing interacts with other design characteristics of the learning environment. In this regard, we take a closer look at the effects of setting because setting may be another characteristic that influences learners’ perceptions of a pedagogical agent’s expertise.

### 1.4. Effects of setting

There is an ongoing debate on whether authentic settings comparable to professional contexts should be used in education and how this could be implemented (Anker-Hansen and Andreé, 2019). To this end, several studies observed that more authentic settings such as research facilities for science topics, positively influenced students’ perceptions of relevance and interest (Betz, 2018; Schüttler et al., 2021).

However, whether the result that an authentic real-world setting improves perceived interest generalizes to better learning outcomes when authentic settings are implemented in learning videos is up to debate. Importantly, this question arises for both photo-realistic videos as well as for digitally animated videos (Schroeder and Adesope, 2012). For photo-realistic videos, the setting is defined as the physical environment in which the learning video was shot (see Merkt et al., 2020). Applying this definition to digitally animated videos, we define setting as the digitally designed background of the video.

To explain possible effects of setting on learning, Merkt et al. (2020) proposed that the setting of the learning video may affect the evaluation of an instructor’s expertise. The learning videos in the study by Merkt et al. (2020) covered floral topics and were either shot in a botanical garden as an authentic setting or in front of a white wall as a neutral setting. Across two experiments, there were no consistent setting effects for learning outcomes, so that the authors come to the cautious conclusion that the effects of setting on learning outcomes may be negligible. Further, contrary to the authors’ initial assumptions in both experiments, there were no effects of setting on the perceived expertise of the instructor. However, Merkt et al. (2020) discussed whether including an inappropriate setting instead of a neutral setting (white wall) as a control condition may result in setting effects and different expertise judgments. Further, it is possible that expertise judgments regarding a virtual pedagogical agent may be more susceptible to external cues because virtual pedagogical agents may provide less cues that learners may use to judge expertise.

In summary, previous research did not find consistent effects of setting on learning outcomes and perceived expertise. The current experiment will therefore address the effect of an appropriate setting compared to an inappropriate setting and additionally address potential interactions with a virtual pedagogical agent’s clothing.

### 1.5. The present research – hypotheses

Both for photo-realistic and for animated videos, producers of instructional materials are confronted with the questions what instructors/pedagogical agents should wear and in which setting the instructional materials should be produced. Based on the presented theoretical framework, it becomes evident that both of these design characteristics may affect the learning process by means of inducing different perceptions of expertise, whereas empirical evidence in favor of these assumptions is mostly mixed. In particular, previous research on clothing does imply that thematically appropriate clothing may be beneficial for learning (Beege et al., 2019; Schmidt et al., 2019), whereas the effects of a thematically appropriate setting are considered to be rather negligible (Merkt et al., 2020). Because potential effects of clothing and setting may be driven by the same underlying mechanism (i.e., perceived expertise), we decided to investigate both of these design factors in the same experiment, employing a 2 × 2-factorial between subject design. Given the lack of research combining these two factors, we decided to treat the interaction of the two factors as an open research question because there is no clear theoretical indication about the potential interplay of these two factors. On the one hand, it may be sufficient to include one cue for instructor expertise, on the other hand, it is also conceivable that the different sources for expertise amplify each other.

Building on these considerations, we preregistered the following hypotheses on OSF.

H1: The video including the pedagogical agent wearing thematically appropriate clothing is rated as more professional than the video including the pedagogical agent wearing thematically inappropriate clothing.

H2: The pedagogical agent wearing thematically appropriate clothing is considered to have a higher expertise than the pedagogical agent wearing thematically inappropriate clothing.

H3: The pedagogical agent wearing thematically appropriate clothing results in better learning outcomes than the pedagogical agent wearing thematically inappropriate clothing.

Based on previous mixed evidence on the effects of setting on learning outcomes (see Merkt et al., 2020), we refrain from formulating hypotheses regarding a main effect of setting. Further, the study explores the interaction of clothing and setting.

## 2. Materials and methods

### 2.1. Participants and design

Overall, 231 participants completed the experiment. Thirty-two participants were excluded due to pre-defined exclusion criteria, namely technical difficulties during the presentation of the video (1 participant), use of search engines such as Google (7 participants), a native language other than German (2 participants), or taking notes during the learning video (21 participants). This resulted in a final sample of 200 German-speaking participants (131 male, 68 female, and 1 diverse), recruited using Prolific. The participants were randomly assigned to one of the four conditions (2 × 2 design) and received 4.38 Pounds for their participation. Their mean age was 29.52 years (*SD* = 8.73). Even though a power analysis revealed that 125 participants would have been sufficient to detect a medium effect ($\eta_{p}^{2}=0.06$) with a predefined alpha level of 0.05 and power of 0.80, we pre-registered to collect data of 200 participants to increase power and identify potential interactions.

Participants were randomly assigned to one of four conditions resulting from a 2 × 2 factorial between-subjects design with the clothing of the pedagogical agent (thematically appropriate or thematically inappropriate clothing) and the setting of the animated video (thematically appropriate or thematically inappropriate setting) as the independent variables. Learning outcomes and different evaluation questions were collected as dependent variables.

### 2.2. Learning materials

**FIGURE 1:**
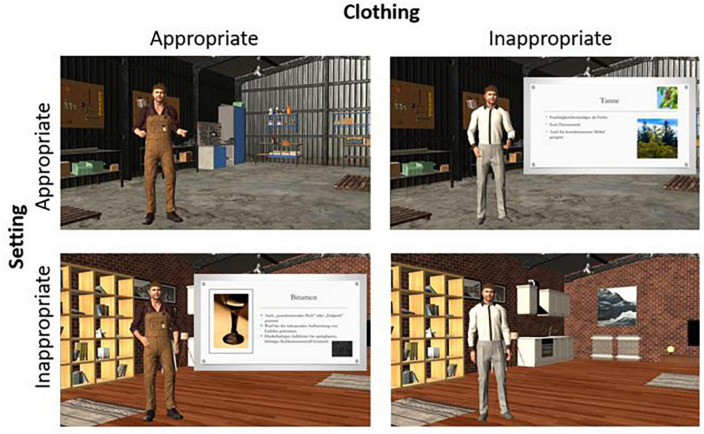
Conditions of the learning video displaying the variation of setting and clothing. To illustrate the insertion of the presentation, one version with and one without the presentation was selected for each setting.

The learning material consisted of an animated video (duration: 19:30 min) that was presented to the participants in one of four different versions, depending on the experimental condition they were assigned to (see Figure 1). In the video, a pedagogical agent presented learning content on craft topics such as wood and its applications, connections, weather protection, sawing, glues, tools, locks, and corrosion. The information was partly visualized in a PowerPoint presentation that was superimposed over the background on the right-hand side of the agent. The presentation appeared only occasionally to visually highlight important content, whereas the setting was fully visible throughout the remainder of the video. The pedagogical agent had a male human voice and could be seen in its entirety (full-body representation) displaying conversational gestures (see Davis, 2018) and mimic. Neither the facial expressions nor the gestures did have any deictic functions. The pedagogical agent was created with Character Creator 3.3 and animated with iClone 7.8.

As appropriate clothing for a craft theme, the pedagogical agent wore brown dungarees. The design of this clothing was based on the recommendations for workwear of the Employer’s Liability Insurance Association for Wood and Metal in Germany (Berufsgenossenschaft Holz und Metall [BGHM], 2019) and can thus be considered prototypical for the cultural context in which the study was conducted. For example, according to a study on woodworking injuries, 68% of professional craftsmen wore safety clothing similar to the equipment described in the above mentioned handout (Loisel et al., 2014). In contrast, in the inappropriate clothing condition, the pedagogical agent wore a white buttoned shirt with neat gray trousers. Since the visual appearance of our setting should appear as if crafts could be carried out without difficulty, the pedagogical agent was placed in a classic workshop setting with a gray concrete floor, tool walls, a workbench, pallets, and garage-like walls in the appropriate setting condition. In contrast, for the inappropriate setting condition, the pedagogical agent was placed in a living room environment with a wooden laminar floor, shelves, paintings, a kitchenette in the background, and small rugs on the floor, where the practical execution of craft activities would be more difficult. The arrangement, as well as the number and size of the objects in the background of both settings were chosen to match as closely as possible. It was ensured that the videos did not differ regarding any other characteristics than clothing and setting, especially because facial expressions, gestures, and speed of delivery could be kept perfectly constant due to the digital nature of the videos.

### 2.3. Measures and instruments

#### 2.3.1. Prior knowledge and interest

Prior knowledge and interest in the learning topics were each measured with 5 items on a 7-point Likert scale in self-assessment (cf. Harp and Mayer, 1997; Kühl et al., 2019; Sondermann and Merkt, 2023), ranging from 1 (almost no prior knowledge/interest at all) to 7 (a lot of prior knowledge/very great interest). The topics covered in these questions were: Areas of use for different types of wood, wood connection, weather protection, tools, and corrosion. Participants’ scores for prior interest and prior knowledge were determined by averaging their ratings for each of the topics. Cronbach’s alphas for the prior knowledge items and the interest items were 0.92 and 0.93, respectively.

#### 2.3.2. Evaluation of the learning video

Following the research of Merkt et al. (2020), participants were asked to rate the video on multiple 7-point Likert scales from 1 (lowest score) to 7 (highest score) covering different video characteristics. These characteristics included expertise of the instructor (“How much of an expert was the instructor?”), professionalism (“How professional did the video appear to be?”), quality of explanations (“How would you rate the quality of the explanations?”), and distraction by the setting (“How distracting was the setting of the video?”). As a manipulation check, we also measured the fit between the agent’s clothing (“How would you rate the fit between the teaching theme and the avatar’s^1^ clothing?”) and the setting (“How would you rate the fit between the teaching topic and the setting of the video?”) to the topics presented. Further, we measured the invested mental effort (“How much effort did you invest in following the contents of the video?”; see Paas, 1992).

Additionally, we measured credibility of the pedagogical agent (“How credible do you perceive the avatar to be?”), naturalness of gestures and facial expressions of the pedagogical agent (“How natural did you find the gestures of the avatar?” and “How natural did you find the facial expressions of the avatar?”), perceived appropriateness of the video length (“How appropriate did you find the length of the video?”), the perceived personal addressing (“It seemed like the lecturer was speaking directly to me”; see Beege et al., 2019) and the perceived difficulty (“How difficult was the content of the video?”).

#### 2.3.3. Knowledge test

The learning outcome was assessed with 20 multiple choice questions with four possible answers, one of which was correct. For each question, participants received one point for selecting the correct answer, resulting in a maximum score of 20 points. Cronbach’s alpha for the knowledge items was 0.73. The items included questions such as “What is the average humidity of free-standing wood without weather protection?” (answer options: 18 vs. 12 vs. 22 vs. 25%, correct answer: 18%).

To assess whether the questions in the knowledge test could be answered based on general knowledge, we piloted the items in a sample of 20 participants (9 female and 11 male). The pilot participants’ mean age was 29.15 years (*SD* = 9.07). The pilot study was run on LimeSurvey and the participants were recruited via Prolific, an online survey platform with a large number of registered participants. They gave informed consent and were instructed to answer the questions solely based on their knowledge without using search engines. Afterward they filled in a knowledge test consisting of 39 multiple choice questions without any previous access to learning materials. From this pool of items from the pilot study, we then selected 20 items for the current study that showed adequate moderate difficulty. The mean score for those 20 items of the knowledge test in the pilot study answered without learning materials was *M* = 7.65 (*SD* = 2.03). Thus, whereas a small proportion of the questions could be answered based on general knowledge, the test cannot be considered too easy and provides room for improvement after watching the learning video.

### 2.4. Procedure

The experiment was run online using LimeSurvey with participants recruited via Prolific. After participants agreed to the consent form, they were randomly assigned to one of four experimental conditions. Afterward, a picture and sound test was administered to ensure that the learning video was correctly presented with picture and sound. Next, prior knowledge and general interest in the learning content was recorded. One of the four versions of the learning video was presented subsequently, according to the experimental condition the participants were randomly assigned to. Following the video, participants were asked to evaluate the video before they filled in the knowledge test. After the knowledge test, the variables regarding the exclusion criteria (technical difficulties during the presentation of the video, use of search engines, a native language other than German, or taking notes) were assessed with an explicit request for honesty and an additional remark that this information was important for assessing data quality but would not affect payment. Finally, demographic data were queried, and the debriefing took place. The procedure was approved by the Local Ethics Committee of the German Institute for Adult Education.

## 3. Results

Data were analyzed with 2 × 2 ANOVAs with the independent variables Clothing (thematically appropriate vs. inappropriate) and Setting (thematically appropriate vs. inappropriate) and the respective outcomes as dependent variables. Further, to test whether the video was suitable for knowledge acquisition, a one sample *t*-test was used to compare the mean score that participants had achieved in the pilot study of the knowledge test (i.e., 7.65 points), in which the items were tested without learning materials, to the mean score achieved in this experiment over all conditions.

The data and the analysis script are available on OSF. Descriptive data are presented in Table 1.

**TABLE 1:** Overall means and standard deviations.

Variable	Appropriate clothing			Inappropriate clothing			Overall		Overall
	**Appropriate setting**	**Inappropriate setting**	**Overall**	**Appropriate setting**	**Inappropriate setting**	**Overall**	**Appropriate setting**	**Inappropriate setting**	
Prior interest	3.29 (1.49)	3.15 (1.29)	3.22 (1.39)	3.43 (1.61)	3.45 (1.60)	3.44 (1.60)	3.36 (1.54)	3.30 (1.46)	3.33 (1.49)
Prior knowledge	2.34 (1.02)	2.19 (1.20)	2.26 (1.11)	2.49 (1.06)	2.56 (1.29)	2.52 (1.18)	2.41 (1.04)	2.37 (1.25)	2.39 (1.15)
Knowledge test	13.14 (4.06)	14.58 (3.50)	13.86 (3.84)	13.96 (3.75)	13.34 (3.23)	13.65 (3.49)	13.55 (3.91)	13.96 (3.41)	13.76 (3.67)
Professionalism	3.80 (1.48)	4.08 (1.62)	3.94 (1.56)	3.78 (1.43)	3.66 (1.72)	3.72 (1.58)	3.79 81.45)	3.87 (1.68)	3.83 (1.57)
Expertise	4.42 (1.72)	4.08 (1.87)	4.25 (1.79)	3.96 (1.76)	3.72 (1.98)	3.84 (1.87)	4.19 (1.75)	3.90 (1.92)	4.04 (1.84)
Quality	5.02 (1.13)	5.30 (1.15)	5.16 (1.19)	5.10 (1.13)	4.96 (1.32)	5.03 (1.23)	5.06 (1.17)	5.13 (1.24)	5.10 (1.21)
Naturalness – gestures	2.44 (1.37)	2.72 (1.39)	2.58 (1.38)	2.46 (1.42)	2.46 (1.49)	2.56 (1.45)	2.45 (1.39)	2.58 (1.44)	2.52 (1.41)
Naturalness – mimic	2.34 (1.17)	2.72 (1.32)	2.53 (1.26)	2.44 (1.45)	2.28 (1.46)	2.36 (1.45)	2.39 (1.31)	2.50 (1.40)	2.45 (1.36)
Difficulty	3.64 (1.36)	3.34 (1.47)	3.49 (1.42)	3.46 (1.41)	3.28 (1.43)	3.37 (1.42)	3.55 (1.39)	3.31 (1.44)	3.43 (1.42)
Effort invested	4.70 (1.54)	4.54 (1.58)	4.62 (1.56)	4.78 (1.94)	4.74 (1.65)	4.76 (1.79)	4.74 (1.74)	4.64 (1.61)	4.69 (1.68)
Credibility	4.52 (1.75)	4.58 (1.86)	4.55 (1.80)	3.78 (1.75)	3.92 (1.94)	3.85 (1.84)	4.15 (1.78)	4.25 (1.92)	4.20 (1.85)
Thematical fit – clothing	6.06 (1.04)	5.82 (0.89)	5.94 (0.97)	3.82 (1.64)	3.74 (1.45)	3.78 (1.55)	4.94 (1.77)	4.78 (1.59)	4.86 (1.68)
Thematical fit – setting	5.56 (1.16)	4.2 (1.55)	4.88 (1.52)	5.16 (1.55)	3.8 (1.68)	4.48 (1.74)	5.36 (1.38)	4.00 (1.62)	4.68 (1.65)
Distraction	2.58 (1.25)	2.96 (1.64)	2.77 (1.46)	2.76 (1.66)	3.32 (1.66)	3.04 (1.68)	2.67 (1.46)	3.14 (1.65)	2.91 (1.57)
Length	3.86 (1.60)	3.68 (1.51)	3.77 (1.56)	3.70 (1.71)	3.78 (1.60)	3.74 (1.66)	3.78 (1.65)	3.73 (1.56)	3.76 (1.60)
Personal address	3.24 (1.57)	3.12 (1.63)	3.18 (1.60)	3.08 (1.77)	2.94 (1.78)	3.01 (1.77)	3.16 (1.67)	3.03 (1.70)	3.09 (1.68)
*N*	50	50	100	50	50	100	100	100	200

### 3.1. Manipulation check

Regarding the perceived appropriateness of the clothing, the thematically appropriate clothing was rated to be more suitable (*M* = 5.94, *SD* = 0.97) for the topic than the thematically inappropriate clothing (*M* = 3.78, *SD* = 1.55), *F*(1,196) = 138.89, *p* < 0.001, $\eta_{p}^{2}=0.41$. There was neither a main effect of the Setting, *F* < 1; nor an interaction Clothing × Setting, *F* < 1.

Regarding the appropriateness of the setting, the thematically appropriate setting was rated as more appropriate (*M* = 5.36, *SD* = 1.38) than the thematically less appropriate setting (*M* = 4.00; *SD* = 1.62), *F*(1,196) = 41.10, *p* < 0.001, $\eta_{p}^{2}=0.17$. There was also neither a main effect of Clothing, *F*(1,196) = 3.56, *p* = 0.061, $\eta_{p}^{2}=0.02$; nor an interaction Clothing × Setting, *F* < 1.

### 3.2. Prior knowledge, interest, and demographics

With regard to self-reported prior knowledge (*M* = 2.39; *SD* = 1.15), there were no main effects of Clothing, *F*(1,196) = 2.56, *p* = *0.111*, $\eta_{p}^{2}=0.01$, and Setting, *F* < 1, and no interaction Setting × Clothing, *F* < 1. Likewise, for the self-reported interest (*M* = 3.33; *SD* = 1.49), there were no main effects of Clothing, *F*(1,196) = 1.07, *p* = 0.302, $\eta_{p}^{2}=0.01$, and Setting, *F* < 1, as well as no interaction Clothing × Setting, *F* < 1. Thus, we can assume that the groups were comparable before the experiment.

Further, neither the proportion of gender *X*^2^ (6, *N* = 200) = 7.50, *p* = 0.277, nor the proportion of highest educational qualifications, *X*^2^ (15, *N* = 200) = 15.46, *p* = 0.419, differed between the groups. With regard to age, there were no main effects of Clothing, *F* < 1, and Setting, *F* < 1, and no interaction Clothing × Setting, *F*(1,196) = 1.49, *p* = 0.224, $\eta_{p}^{2}=0.01$. Thus, we can assume that the groups were comparable regarding demographic criteria.

### 3.3. Perceived professionalism and expertise

Regarding the first two hypotheses, the analyses revealed no significant differences between the conditions. For perceived professionalism of the video (*M* = 3.83, *SD* = 1.57), there were no main effects of Clothing and Setting and no significant interaction Clothing × Setting, all *F* < 1. Also, concerning perceived expertise of the agent (*M* = 4.04; *SD* = 1.84), there were no significant main effects of Clothing, *F*(1,196) = 2.49, *p* = 0.116, $\eta_{p}^{2}=0.012$, and Setting, *F*(1,196) = 1.25, *p* = 0.266, $\eta_{p}^{2}=0.01$, and no significant interaction Clothing × Setting, *F* < 1.

### 3.4. Learning outcomes

For learning outcomes, there were no significant main effects of either Clothing or Setting, both *F* < 1. However, there was an interaction Clothing × Setting, *F*(1,196) = 3.98, *p* = 0.047, $\eta_{p}^{2}=0.02$. Following up on this interaction, Bonferroni-adjusted *post-hoc* comparisons revealed that there was no significant effect of setting for the inappropriate clothing *F* < 1, whereas in the appropriate clothing condition, the inappropriate setting (*M* = 14.58, *SD* = 3.50) resulted in slightly better learning outcomes than the appropriate setting (*M* = 13.14, *SD* = 4.06), *F*(1,196) = 3.89, *p* = 0.050, $\eta_{p}^{2}=0.02$. When the interaction was resolved the other way, the Bonferroni-adjusted *post-hoc* tests showed no effect of clothing, neither for the appropriate setting, *F*(1,196) = 1.26, *p* = 0.263, $\eta_{p}^{2}=0.006$, nor for the inappropriate setting, *F*(1,196) = 2.89, *p* = 0.091, $\eta_{p}^{2}=0.015$.

Further, results of a one sample *t*-test showed that participants in all conditions (*M* = 13.76; *SD* = 3.67) learned from the video because their mean score was higher than the mean score of participants in the pilot study (i.e., *M* = 7.65) that had answered the same items without the learning material, *t*(199) = 23.55, *p* < 0.001.

### 3.5. Further evaluation variables

For distraction, there was a significant main effect of Setting, revealing that the inappropriate setting (*M* = 3.14, *SD* = 1.65) was rated to be more distracting than the appropriate setting (*M* = 2.67, *SD* = 1.46), *F*(1,196) = 4.53, *p* = 0.035, $\eta_{p}^{2}=0.02$. There was no main effect of Clothing, *F*(1,196) = 1.49, *p* = 0.223, $\eta_{p}^{2}=0.01$; and no interaction Clothing × Setting, *F* < 1.

For credibility, there was a main effect of Clothing, showing that the appropriately dressed pedagogical agent was rated as more credible (*M* = 4.55, *SD* = 1.80) than the thematically inappropriately dressed agent (*M* = 3.85, *SD* = 1.84), *F*(1,196) = 7.31, *p* = 0.007, $\eta_{p}^{2}=0.04$. There was no main effect of Setting, *F* < 1, and no interaction Clothing × Setting, *F* < 1.

There were no main effects or interactions of Clothing and Setting in terms of perceived difficulty, facial expressions of the pedagogical agent, naturalness of gestures, mental effort invested, quality of explanations, perceived appropriateness of the video length and perceived personal addressing, all *F* < 1.44, all *p* > 0.159.

## 4. Discussion

In this experiment, we investigated the effects of a pedagogical agent’s clothing and the setting of an animated video on learning outcomes and participants’ subjective evaluations such as perceived agent expertise and professionalism of the video. Overall, the experiment did not provide any strong evidence for systematic effects of a pedagogical agent’s clothing and the setting of an animated learning video. Contrary to our expectations, the thematically appropriate clothing did not result in more perceived professionalism of the video (Hypothesis 1) or perceived expertise of the agent (Hypothesis 2) than the thematically inappropriate clothing, even though the manipulation check clearly showed that the thematically appropriate clothing was perceived as more appropriate and that the appropriately dressed agent was perceived as more credible than the inappropriately dressed agent. In this regard, it should be mentioned that even though the inappropriate clothing resulted in lower appropriateness and credibility judgments, the inappropriate (i.e., formal) clothing was still judged to be moderately appropriate (*M* = 3.78 on a scale ranging from 1 to 7) and the agent was judged to be moderately credible (*M* = 3.85 on a scale ranging from 1 to 7) in the formal clothes. A possible explanation for this could be that both thematically appropriate clothing and formal clothing exude expertise to the same extent, as formal clothing may be associated with expertise in different fields. Whereas we used formal clothing because we considered it to be inappropriate in a handicraft context, future studies may want to contrast thematically appropriate clothing with clothing that is usually strongly associated with other professions. In the context of a handicraft topic, white coats or police uniforms could be examples for highly inappropriate clothing since they might be strongly associated with other occupations.

Further, contrary to Hypothesis 3, appropriate clothing did not lead to better learning outcomes than the formal clothing in the inappropriate condition.

Regarding the open research question concerning the effect of setting on the learning outcomes, we did not find any setting effects, corroborating cautious conclusions from previous research that there may be no overall setting effects (see Merkt et al., 2020). However, similar to the issues discussed regarding our manipulation of clothing, the living room setting in the inappropriate setting condition was rated to be moderately appropriate (score of 4.00 on a scale ranging from 1 to 7), so that we may have succeeded in manipulating the level of appropriateness but did not manage to provide learners with an inappropriate setting.

However, an interaction between clothing and setting may shed light on some potential boundary conditions for setting effects. In particular, the inappropriate setting resulted in better learning outcomes than the appropriate setting when the pedagogical agent wore appropriate clothing, whereas there was no setting effect when the pedagogical agent wore inappropriate clothing. The result that the appropriate setting did not lead to better learning regardless of the clothing could be explained with the expectancy violations theory (Burgoon, 1993; Dunbar and Segrin, 2012). The appropriate setting, especially combined with the appropriate clothing of the agent may have created the false expectation that something practical would be demonstrated on the machines depicted in the background of the authentic setting. These unmet expectations may have been detrimental to the learning outcomes. In this experiment, the pedagogical agent did not demonstrate how to use the authentic machines because we aimed at keeping the learning content constant across the appropriate and the inappropriate setting conditions. Therefore, interactions with the machines could not be included since the machines were only present in half of the conditions. To determine whether the violation of expectations played a crucial role, participants in future experiments could be asked how they imagine an expert in the field and what they expect from the learning materials before the learning phase. Further, because this unexpected effect is very small ($\eta_{p}^{2}=0.02$), it should be replicated before jumping to premature conclusions.

Finally, the rather long duration of the video (19:30 min) may have leveled out potential clothing and setting effects because these may wear off over time, as learners may pay more attention to clothing and setting at the beginning of the video. In particular, Guo et al. (2014) reported that general attention while watching videos significantly decreases after 6 min. Since clothing and setting did not change throughout the video, it could have faded into the background of attention while participants watched the video for approximately 20 min. Testing video length as a potential boundary condition for clothing and setting effects may thus be an interesting pathway for future research. These studies could additionally use process data such as eye-tracking to determine whether the attention wears off over time.

### 4.1. Limitations

The results are subject to some limitations. First, we tested the effect of clothing and setting with only one specific exemplar for appropriate and inappropriate manifestations. However, looking at clothing, craft clothing can include a wide range of clothing, especially as many sub-disciplines have their own specific clothing and safety equipment (such as painters and varnishers). Thus, the generalizability of these findings should be tested for more sub-disciplines in future experiments. Further, the subject areas should be extended to other occupational groups, as many other professions also have distinctive professional clothing that could be strongly associated with expertise (e.g., doctors, nurses, cooks, security guards, flight attendants, and pilots). Similar limitations should be considered for the setting. The range of settings potentially perceived as appropriate could be narrower or wider depending on the occupational group. Craft topics have a rather wide range, as for example, car repair shops look very different from carpentry shops. Regarding these factors, future research questions in this area should be chosen accordingly.

Further, it should be noted that the interpretation of our findings regarding specific pieces of clothing and the setting as depicted in Figure 1 is limited to the cultural background of the study (i.e., Germany). In particular, the appropriateness of different pieces of clothing and different types of setting for different purposes may vary with culture. However, it should also be noted that the investigated underlying principle of thematical appropriateness should be more generalizable across cultures if researchers from different cultures carefully select pieces of clothing and a setting that is considered appropriate for a specific topic in their culture. Further, the generalizability of these findings across cultures could also be tested to see whether there are different effects of the same learning materials in different cultures.

Whereas the use of different types of clothing to evoke different perceptions of expertise could be considered a problematic case of stereotyping, we want to stress that this research does not endorse the use of stereotyping to manipulate learners. Therefore, this study did not use exaggerated stereotypical clothing, but rather prototypical clothing which is usually worn by persons of a specific occupation (Berufsgenossenschaft Holz und Metall [BGHM], 2019). Because some types of clothing may be closely linked to specific occupations (see Kodžoman, 2019), we were interested whether learners use these associations between clothing and specific occupations as a heuristic to determine a pedagogical agents’ expertise. If such effects had occurred, it would have been an interesting follow up question how to handle and prevent such effects.

Further, since the elaboration likelihood model assumes that heuristics are often used as a shortcut instead of more elaborated cognitive processes (Petty and Cacioppo, 1986), it is feasible to assume that learners may rely on heuristics more heavily when their cognitive load is high. However, since it was not the aim of our study to investigate whether cognitive load influenced the usage of heuristics for evaluating the expertise of a pedagogical agent, we did not measure cognitive load specifically, but decided to use the item by Paas (1992) to gain insights on the effects of mental effort. Since cognitive load and mental effort are distinct concepts that affect learning outcomes in different ways (see Schnaubert and Schneider, 2022), the question whether heuristics play an increased role under high cognitive load cannot be answered with this experiment and should therefore be addressed in further research that measures cognitive load using more nuanced instruments that assess different facets of cognitive load (Klepsch et al., 2017; Krieglstein et al., 2023).

## 5. Conclusion

This experiment tried to shed light on the question whether it is necessary to design and animate thematically appropriate clothing for pedagogical agents and add an appropriate setting to an animated video to increase learning outcomes and perceived expertise of the agent as well as perceived professionalism of the learning video. The results imply that in case of a handicraft topic, designers of animated videos may have some degrees of freedom when selecting the clothing of the agent and the setting of the video because participants in all conditions acquired knowledge and the influence of clothing and setting on learning outcomes and perceived expertise was negligible. However, it should be noted that none of the clothing and setting in our study was perceived to be inappropriate for the learning materials. Thus, the use of a highly inappropriate clothing and setting may still constitute a boundary condition for clothing and setting effects.

## Data availability statement

The datasets presented in this study can be found in online repositories. The names of the repository/repositories and accession number(s) can be found below: OSF – https://osf.io/qm4pv/?view_only=1715aaa02cb74e588b591882b08a3bd3.

## Ethics statement

The studies involving humans were approved by the Local Ethics Committee of the German Institute for Adult Education – Leibniz Center for Lifelong Learning e.V. The studies were conducted in accordance with the local legislation and institutional requirements. The participants provided their written informed consent to participate in this study.

## Author contributions

DD: writing—original draft, methodology, editing, conceptualization, and data analysis. MM: writing—reviewing, conceptualization, and supervision. Both authors contributed to the article and approved the submitted version.

## Footnotes

1 Since our study took place in Germany, where the term “pedagogical agent” is not very common, we decided to refer to our pedagogical agent as avatar in the evaluation questions to avoid misunderstandings among the participants.
